# Contributors to Gender Disparities in Parkinson’s Disease Caregiving

**DOI:** 10.1177/08919887251329957

**Published:** 2025-03-21

**Authors:** Sarah Horn, Yunfeng Dai, Samuel S. Wu, Nabila Dahodwala

**Affiliations:** 1 Department of Neurology, 14742 The University of Texas Health Science Center at San Antonio, San Antonio, TX, USA; 2 Department of Biostatistics, 3463 University of Florida, Gainesville, FL, USA; 3 Department of Neurology, Perelman School of Medicine, 14640 University of Pennsylvania, Philadelphia, PA, USA

**Keywords:** Parkinson’s disease, caregiving, health inequities, gender

## Abstract

**Background:**

Women with Parkinson’s disease (PD) are less likely to have a caregiver.

**Objective:**

To determine factors contributing to gender disparities in PD caregiving.

**Methods:**

We conducted a cross-sectional survey of people with PD and caregivers participating in the Parkinson’s Foundation Parkinson’s Outcomes Project and compared patient and caregiver characteristics by gender.

**Results:**

Among PD patients, 20.7% of 1663 women and 14.2% of 3005 men had no caregiver (*P* < 0.001). Women without caregivers were older (69.1 vs 66.3, *P* < 0.001), less likely to be married (30.4% vs 54.7%, *P* < 0.001), and more likely to be taking an antidepressant (41.8% vs 30.9%, *P* = 0.002) than men. Using stepwise logistic regression models, gender differences in access to caregiving were explained by marital status. Among caregivers, women reported more strain (*P* < 0.001) and had less time for other family members (*P* < 0.001).

**Conclusion:**

Fewer women with PD have caregivers because they are less likely to have a spouse.

## Plain Language Summary

Caregivers play a vital role in the lives and health of people with Parkinson’s disease (PwP). However, women with Parkinson’s disease (PD) are less likely to have a caregiver and are more likely to be a caregiver themselves. Furthermore, women caregivers for PwP are more likely than men to feel burnout due to caregiving. This study examines potential reasons for these gender-related differences in PD caregiving. To better understand the experiences of caregivers, surveys were given to caregivers of PD patients who receive care at Parkinson’s Foundation Centers of Excellence in the United States. Our findings show that women without caregivers are older, less likely to be married, and have higher rates of depression. Marriage is by far the strongest predictor for having a caregiver. Among PD caregivers, women (1) care for PwP who are more disabled, (2) have more burnout, and (3) sacrifice more of their time with other family members. More research is needed to identify ways to better support PwP and their caregivers.

## Introduction

Parkinson’s disease (PD) causes impairment in motor function as well as numerous non-motor symptoms such as dementia, depression, and psychosis. The motor and non-motor features collectively contribute to disability, particularly with advancing disease.^1,2^ People with Parkinson’s disease (PwP) need more help from others as the disease progresses, and informal (unpaid) caregivers are integral to the care of PwP.^3,4^ By late-stage PD, patients have complete loss of independence and are very dependent on caregivers for daily functioning.^5^ Caregivers are an understudied part of the Parkinson’s disease care team, and more research is needed regarding caregiver responsibilities and needs.

There are 53 million informal caregivers in the United States.^6^ They provide most of the care for individuals with serious illness living in the community and access to informal caregivers improves outcomes and delays time to institutionalization.^7-9^ Among PwP, caregivers are instrumental in tasks such as getting patients to appointments, managing medications, and reporting new issues to the medical team. However, caregivers typically have few resources and support to aid in this important task.^10^

Furthermore, significant gender differences in caregiving exist. Disabled women are less likely to receive informal caregiving and have higher rates of nursing home placement than men.^11,12^ Women have less access to caregivers at the end-of-life and gender disparities permeate numerous aspects of end-of-life care. Women are more likely to outlive their spouses and live alone at the end of life, with fewer informal caregivers and resources left to care for them. Women are also more likely to accept formal care to avoid being a burden, a sentiment potentially influenced by prior life experience as a caregiver themselves.^13^ Similarly, women with PD have less access to informal caregivers, with 88.4% of men vs 79.4% of women having a caregiver.^14^ Women with PD are also more likely to hire paid caregivers and are more likely to be caregivers themselves; moreover, women caregivers report higher caregiver strain.^14^ The mechanisms for these gender disparities are unknown though prior research suggests that differences in caregiver demographics may contribute.^11^ Across cultures and health conditions, women provide most of the informal care for people with chronic medical conditions, and women may adopt this caregiving role due to societal and cultural expectations and demands traditionally placed on women.^6,15,16^ Most caregivers of elderly disabled patients in general are women, with children as the dominant caregivers for disabled women and wives as the dominant caregivers of disabled men.^11,17^

Determining contributors to gender disparities in PD caregiving can identify areas amenable to intervention and inform healthcare policy. We evaluated potential contributors to gender disparities by surveying PwP and their caregivers.

## Materials and Methods

### Standard Protocol Approvals and Patient Consents

The protocol was approved by the Institutional Review Board at each site prior to participant recruitment and written informed consent was obtained from both PwP and caregivers. Caregivers who participated in the additional sub-study signed an additional written informed consent form.

### Study Design and Sample

We surveyed caregivers of PwP using participants drawn from the Parkinson’s Foundation Parkinson’s Outcomes Project (PF-POP), which is an ongoing, prospective, observational study of PD that annually assesses health outcomes among participants with PD and their caregivers. The study is conducted at 21 PF Centers of Excellence located in Canada, the Netherlands, Israel, and the United States.^18^ Participant enrollment began in 2009, and new participants continued to be enrolled every year through 2023. For this cross-sectional sub-study, a caregiver survey was sent to caregivers of participants at 16 centers in the United States. Surveys were distributed at the discretion of each site by mail, email, and/or in person. A single caregiver was identified by each study participant. Completed surveys were collected during routine study visits from 2016-2018, providing a convenience sample among PF-POP participants. All patients seen at these participating sites for regular PD care were eligible to participate in the study. The primary goal of the study was exploratory, that is, to uncover patterns and relationships.

### Measures

The PF-POP collects annual patient and caregiver data from study participants. The caregiver sub-study survey collected self-reported data on caregivers including basic information about the caregiver and patient, amount and type of caregiving provided, Center for Epidemiological Studies Depression Scale (CES-D),^19,20^ caregiver preparedness scale,^21,22^ support systems in place, specific needs of the PD patient, Multidimensional Caregiver Strain Index (MCSI),^23^ Movement Disorder Society-sponsored revision of the Unified Parkinson’s Disease Rating Scale (MDS-UPDRS) Part II,^24^ and overall caregiving experience. Most questions were close-ended, with the exception of one open-ended question. Participants were asked to self-identify their gender by selecting either “Male” or “Female” on the survey. The survey (excluding the standardized questionnaires) is provided as Supplemental data. We used the STROBE cross sectional checklist when writing our report.^25^

### Analysis

Descriptive statistics were used to characterize the participants and quantify any missing data. Next, t tests were used for continuous variables with normally distributed data, Wilcoxon rank-sum tests for non-normally distributed continuous data, and χ^2^ tests for categorical variables, to compare demographic and caregiving characteristics by gender of caregivers and patients. The ANOVA test was used to compare normally distributed continuous data with greater than 2 categories and the Kruskal-Wallis test was used to compare non-normally distributed continuous data with greater than 2 categories. Individuals with missing gender data were omitted from analyses. Bonferroni correction was used to correct for multiple comparisons for the following analyses: (1) comparing baseline characteristics of PwP with and without caregivers, corrected *P*-value was 0.005; (2) comparing demographic data between men and women without caregivers, corrected *P*-value was 0.002; (3) comparing demographic data between men and women caregivers, corrected *P*-value was 0.005; and (4) comparing clinical data between men and women caregivers, corrected *P*-value was 0.003. Patient comparisons by caregiver gender were viewed as exploratory and a *P*-value of 0.05 was used. Once univariable correlations were determined, numerous significant individual variables were incorporated into a stepwise multiple logistic regression by iteratively adding meaningful groups of predictors, in order to determine which variables were the most important predictors of the gender disparity in access to a caregiver.

## Results

Among the overall baseline cohort of 4511 women and 7757 men with PD enrolled across all centers in the PF-POP study, 1663 women and 3005 men with PD were seen between 2016-2018 at the 16 US sites participating in the caregiver survey sub study, and a convenience sample of 656 caregivers of PwP completed the caregiver survey. Among 4236 participants with available gender and marital status data, 1177 (81.5%) women and 2568 (92.0%) men with PD were married or in a domestic partnership, and 117 (8.1%) women and 44 (1.6%) men were widowed (*P* < 0.001). The remaining participants were either single (never married) or divorced/separated. Baseline characteristics of the PwP with caregivers who participated in the caregiver sub-study are shown in Table 1 and compared to PF-POP participants with caregivers who did not participate and those without caregivers.

**Table 1.** Baseline Characteristics of PwP With and Without Caregivers.

Patient Characteristics	Has Caregiver Who Completed the Survey (N = 656)	Has Caregiver, did Not Complete Survey (N = 3251)	Has No Caregiver (N = 774)	*P*-Value
Mean age (SD)^a^	68.9 (8.5)	68.7 (9.1)	67.6 (9.6)	0.004
Female, N (%)^b^	199 (30.3)	1119 (34.5)	345 (44.7)	0.000
Race, N (%)^c^				0.002
White	628 (96.2)	3013 (94.0)	705 (93.1)	
Black	4 (0.6)	53 (1.7)	24 (3.2)	
Asian	12 (1.8)	73 (2.3)	8 (1.1)	
Other	9 (1.4)	66 (2.1)	20 (2.6)	
Ethnicity, N (%)^c^				0.001
Hispanic	26 (4.0)	235 (7.6)	39 (5.4)	
Non-hispanic	622 (96.0)	2876 (92.4)	681 (94.5)	
Some college or more education, N (%)^d^	517 (84.5)	1861 (81.1)	558 (88.6)	0.001
Married or domestic partnership, N (%)^e^	607 (93.1)	2929 (94.4)	218 (44.1)	<0.001
Spouse is caregiver, N (%)^f^	592 (91.9)	3103 (95.4)	N/A	<0.001
Living situation, N (%)^g^				<0.001
At home or with family	606 (92.4)	1508 (96.4)	463 (93.5)	
Assisted living or skilled care	17 (2.6)	49 (3.1)	23 (4.6)	
H + Y stage, N (%)^c^				<0.001
1 and 2	360 (56.4)	1916 (63.0)	536 (75.1)	
3	194 (30.4)	820 (27.0)	151 (21.1)	
4 and 5	84 (13.2)	304 (10.0)	27 (3.8)	
Mean PD duration (SD)^h^	10.4 (6.0)	9.2 (6.3)	8.0 (5.8)	<0.001

SD: standard deviation; N: number; N/A: not applicable; H + Y: Hoehn and Yahr

Corrected *P*-value for statistical significance = 0.005 (using Bonferroni correction accounting for 10 comparisons)

^a^Among patients with data available (656 survey completers, 3247 who did not complete survey and have a caregiver, 771 without caregiver).

^b^Among patients with data available (656 survey completers, 3240 who did not complete survey and have a caregiver, 772 without caregiver).

^c^Among patients with data available.

^d^Among patients with data available (612 survey completers, 2295 who did not complete survey and have a caregiver, 630 without caregiver).

^e^Among patients with data available (652 survey completers, 3103 who did not complete survey and have a caregiver, 494 without caregiver).

^f^Among patients with data available (644 survey completers and 3251 who did not complete survey and have a caregiver).

^g^Among patients with data available (656 survey completers, 1564 who did not complete survey and have a caregiver, 495 without caregiver).

^h^PD duration = years since PD motor symptom onset; among patients with data available (644 survey completers, 3228 who did not complete survey and have a caregiver, 769 without caregiver).

### Demographic Data of PwP without Caregivers

Among the PwP enrolled in the PF-POP, 345 (20.7%) women and 427 (14.2%) men reported having no caregiver (*P* < 0.001). Characteristics of the women and men without a caregiver are shown in Table 2. Women without caregivers are older (*P* < 0.001), less likely to be married or in a domestic partnership (*P* < 0.001), and more likely to be taking an antidepressant medication (*P* = 0.002) than men without caregivers. Women perform better than men on a 5-word delayed recall test (<0.001), though both genders perform similarly on a verbal fluency test. There are no differences in race, ethnicity, living situation, Hoehn and Yahr (H + Y) stage, duration of disease, PD medication use, mobility, falls, or hospitalizations between men and women without caregivers (corrected *P*-value for statistical significance is 0.002).

**Table 2.** Characteristics of Female and Male PwP Enrolled in the PF-POP Who Have No Regular Caregiver.

Patient Characteristics	Female Patients (N = 345)	Male Patients (N = 427)	*P*-Value
Mean age (SD)^a^	69.1 (9.2)	66.3 (9.7)	<0.001
Race, N (%)^b^			0.246
White	318 (94.1)	385 (92.3)	
Black	13 (3.8)	11 (2.6)	
Asian	1 (0.3)	7 (1.7)	
Other	6 (1.8)	14 (3.4)	
Ethnicity, N (%)^b^			0.227
Hispanic	13 (4.0)	25 (6.4)	
Non-hispanic	315 (96.0)	365 (93.4)	
Some college or more education, N (%)^b^	249 (90.2)	307 (87.2)	0.023
Marital status, N (%)^b^			<0.001
Has spouse/partner	65 (30.4)	152 (54.7)	
Widowed	54 (25.2)	26 (9.4)	
Living situation, N (%)^b^			
At home or with family	194 (91.1)	267 (95.4)	0.091
Assisted living or skilled care	15 (7.0)	8 (2.9)	
Other	4 (1.9)	5 (1.8)	
Employment status, N (%)^b^			0.025
Full time	35 (16.4)	74 (26.5)	
Part time	23 (10.7)	24 (8.6)	
Not employed	156 (72.9)	181 (64.9)	
H + Y stage, N (%)^b^			0.256
1 and 2	228 (72.4)	306 (77.1)	
3	74 (23.5)	77 (19.4)	
4 and 5	13 (4.1)	14 (3.5)	
Years since symptom onset, mean (SD)^c^	7.9 (5.6)	8.1 (6.0)	0.781
Number of comorbidities, mean (SD)	2.0 (2.0)	1.7 (1.8)	0.147
Verbal fluency, mean animals/min (SD)^d^	20.8 (6.7)	19.8 (6.0)	0.035
Delayed recall, mean (SD)^e^	3.8 (1.2)	3.5 (1.3)	<0.001
TUG, mean seconds (SD)^f^	12.6 (6.7)	11.8 (6.1)	0.079
Fall frequency, N (%)^b^			0.387
None or rare	294 (87.0)	372 (89.6)	
Weekly or monthly	40 (11.8)	38 (9.2)	
Daily	4 (1.2)	5 (1.2)	
Mean # hospitalizations in past year (SD)^g^	0.26 (0.7)	0.24 (0.6)	0.744
Had ED visit in past year, N (%)^h^	96 (30.0)	89 (22.3)	0.020
Medications, N (%)^i^			
Antidepressant	141 (41.8)	129 (30.9)	0.002
Anticholinergic	8 (2.4)	20 (4.8)	0.084
Cognitive enhancer	9 (2.7)	22 (5.3)	0.078
PDQ-39 summary index, mean (SD)^j^	20.3 (14.7)	20.5 (14.9)	0.824

SD: standard deviation; N: number; H + Y: Hoehn and Yahr; TUG: Timed Up & Go test; ED: emergency department; PDQ: Parkinson’s Disease Questionnaire

Corrected *P*-value for statistical significance = 0.002 (using Bonferroni correction accounting for 25 comparisons).

^a^Among patients with data available (343 women and 426 men).

^b^Among patients with data available.

^c^Among 342 women and 425 men.

^d^Among 333 women and 407 men.

^e^Among 337 women and 410 men; delayed recall = number of words recalled from a list of 5.

^f^Among 245 women and 275 men.

^g^Among 325 women and 399 men.

^h^Among 320 women and 398 men.

^i^There were also no differences in male vs female PD patients without a caregiver taking levodopa (*P* = 0.544), a monoamine oxidase B inhibitor (*P* = 0.788), a COMT (catechol-O-methyltransferase) inhibitor (*P* = 0.337), a dopamine agonist (*P* = 0.626), or amantadine (*P* = 0.935); among patients with data available.

^j^Among 327 women and 411 men.

### Demographic Data of PwP with Caregivers

Among the PwP enrolled in the PF-POP, 1317 women and 2575 men reported having a caregiver. Age is similar between men (mean 68.7 ± 8.8) and women (mean 68.6 ± 9.3) with caregivers (*P* = 0.621). Men with caregivers are more likely to be married (95.5% vs 90.3%) and less likely to be widowed (0.7% vs 5.1%) than women with caregivers (*P* < 0.001). While most caregivers are spouses for both men (96.8%) and women (91.1%), women are more often cared for by other relatives (6.5% vs 2.0%) or paid providers (2.2% vs 0.8%, *P* < 0.001). Men with caregivers are more likely to live at home or with family (95.8% vs 93.9%) and less likely to live in a skilled care or assisted living facility (2.4% vs 4.3%) than women with caregivers (*P* = 0.046). Women with caregivers have had PD longer than men (mean 9.8 ± 6.4 vs 9.2 ± 6.1 years, *P* = 0.008) and have poorer quality of life (Parkinson’s Disease Questionnaire (PDQ-39) Summary Index 27.1 ± 17.4 vs 24.0 ± 15.6, *P* < 0.001). There are no differences in race (*P* = 0.493), ethnicity (*P* = 0.147), number of co-morbidities (*P* = 0.316), or hospitalizations (*P* = 0.280) between men and women with caregivers.

### Stepwise Multiple Logistic Regression

In stepwise multiple logistic regression modeling (Table 3) that incorporates variables significantly associated with caregiving in univariable correlations, the impact of gender on caregiver presence remains significant when demographic data (age, race, education level, and employment status), health conditions (delayed recall score, emergency department visit in the past year, and antidepressant use), and PD severity (PDQ-39 Summary Index, H + Y stage, and PD duration) are added to the model (p_gender_ <0.001 for all). Once marital status is added to the model (OR 16.5, *P* < 0.001), gender is no longer a significant predictor of caregiver presence.

**Table 3.** Stepwise Logistic Regression Models and Odds Ratios (95% CI).

	Model 1	Model 2	Model 3	Model 4	Model 5
Female gender (ref: male)	**0.63 (0.54-0.74)**	**0.63 (0.51-0.78)**	**0.61 (0.49-0.77)**	**0.61 (0.48-0.78)**	1.00 (0.75-1.34)
Age		1.01 (0.995-1.02)	1.01 (0.998-1.02)	1.01 (0.999-1.03)	**1.03 (1.01-1.04)**
Race (white)		1.47 (0.53-4.11)	1.72 (0.60-4.94)	1.98 (0.66-5.93)	1.31 (0.38-4.56)
Some college or more education (ref: HS diploma or less)		**0.47 (0.33-0.67)**	**0.50 (0.35-0.73)**	**0.52 (0.35-0.77)**	**0.48 (0.31-0.74)**
Employed (ref: unemployed)		**0.61 (0.48-0.78)**	**0.60 (0.46-0.78)**	**0.74 (0.56-0.98)**	0.78 (0.57-1.08)
ED visit in past year (ref: no recent ED visit)			1.07 (0.83-1.37)	0.94 (0.72-1.23)	1.04 (0.76-1.42)
Memory (5-word delayed recall)			1.05 (0.97-1.14)	**1.12 (1.02-1.22)**	**1.12 (1.01-1.25)**
Antidepressant use			1.17 (0.93-1.48)	1.00 (0.77-1.29)	1.10 (0.82-1.47)
PDQ-39 summary index				**1.01 (1.00-1.02)**	**1.02 (1.01-1.03)**
PD duration				**1.04 (1.01-1.07)**	**1.05 (1.02-1.08)**
H + Y stage 3 or greater				**1.49 (1.08-2.05)**	**1.68 (1.16-2.42)**
Has spouse/partner					**16.5 (12.14-22.52)**

HS: high school; PDQ: Parkinson’s Disease Questionnaire; PD duration: years since motor symptom onset. Statistically significant results (*P* < 0.05) are in bold.

Model 1: gender alone.

Model 2: gender and demographic data.

Model 3: gender, demographics, and health conditions.

Model 4: gender, demographics, health conditions, and PD severity.

Model 5: gender, demographics, health conditions, PD severity, and marital status.

### Demographic Data of Caregivers

Caregivers for 199 women and 457 men with PD completed the additional survey. Caregivers are of the opposite gender for 84.9% of women and 96.5% of men with PD. Among respondents, four (0.6%) were paid caregivers, the remaining were informal caregivers. Characteristics of female and male caregivers are shown in Table 4. Female caregivers are younger than male caregivers (*P* < 0.001; corrected *P*-value threshold for significance is 0.005). Female and male caregivers have a similar employment status (*P* = 0.194), work similar hours per week outside the home (*P* = 0.018), and spend a similar amount of time per week providing care (*P* = 0.942).

**Table 4.** Characteristics of Caregivers Who Participated in the Caregiver Survey Sub-Study.

Caregiver Demographics	Female Caregivers (N = 471)	Male Caregivers (N = 185)	*P*-Value
Mean age (SD)^a^	65.0 (9.1)	67.8 (10.7)	<0.001
Race, N (%)^b^			0.166
White	449 (96.6)	172 (95.0)	
Black	7 (1.5)	1 (0.6)	
Asian	8 (1.7)	5 (2.8)	
Other	1 (0.2)	3 (1.7)	
Ethnicity, N (%)^b^			0.091
Hispanic	9 (2.2)	8 (4.9)	
Not hispanic	392 (97.8)	154 (95.1)	
Relationship to PD patient, N (%)^b^			0.575
Spouse	428 (92.2)	167 (91.8)	
Child (or child-in-law)	21 (4.5)	11 (6.0)	
Parent	5 (1.1)	2 (1.1)	
Other	10 (2.2)	2 (1.1)	
Also provides care to others, N (%)^b^	82 (18.1)	13 (7.3)	0.001
Some college or more education, N (%)	409 (86.8)	157 (84.9)	0.796
Married or domestic partnership, N (%)	453 (96.2)	178 (96.2)	0.446
Financial status, N (%)^b^			
Some money left over	371 (80.3)	153 (83.2)	0.193
Just enough to make ends meet	78 (16.9)	30 (16.3)	
Not enough to make ends meet	13 (2.8)	1 (0.5)	
Currently employed, N (%)^c^	148 (32.0)	67 (37.4)	0.194
Hours/week worked outside the home, mean (SD)	32.1 (17.9)	36.1 (16.2)	0.018
Hours/week providing care, median (IQR)^d^	35 (10.5, 140)	35 (9, 144)	0.942

SD: standard deviation; N: number; IQR: interquartile range. Corrected *P*-value for statistical significance = 0.005 (using Bonferroni correction accounting for 11 comparisons).

^a^Among caregivers with data available (468 women, 184 men).

^b^Among caregivers with data available.

^c^Among 462 women and 179 men.

^d^Among 357 women and 139 men.

### Differences in Patient Characteristics by Caregiver Gender

Female caregivers care for patients with greater disability (mean MDS-UPDRS Part II score 28.0 vs 23.9, *P* < 0.001). There are no differences in overall caregiving activities between men and women caregivers (mean activity help score 33.2 ± 11.3 for female caregivers vs 32.9 ± 11.34 for male caregivers, *P* = 0.494). However, male caregivers provide more assistance with mobility, both inside the house (mean help score 1.76 ± 1.08 vs 1.96 ± 1.10, *P* = 0.035) and outside the house (mean help score 1.87 ± 1.12 vs 2.15 ± 1.19, *P* = 0.004), while female caregivers spend more time doing chores inside the house, for example laundry (mean help score 2.93 ± 1.24 vs 2.54 ± 1.20, *P* < 0.001) and managing finances (mean help score 2.71 ± 1.31 vs 2.41 ± 1.33, *P* = 0.009).

PwP with female caregivers score worse on cognitive testing, including verbal fluency (*P* = 0.031) and delayed recall (*P* < 0.001). Female caregivers are more likely to care for PwP taking a cognitive enhancing medication (*P* = 0.002). Further information about PwP cared for by male vs female caregivers can be found in Table 5. *P*-value threshold was kept at 0.05 as an exploratory analysis.

**Table 5.** Differences in Patient Characteristics by Caregiver Gender.

Patient Characteristics	Female Caregivers (N = 471)	Male Caregivers (N = 185)	*P*-Value
Mean age (SD)	69.2 (8.3)	68.2 (8.9)	0.288
Gender, N (%)			0.000
Female	30 (6.4)	169 (91.4)	
Male	441 (93.6)	16 (8.6)	
Race, N (%)^a^			0.271
White	451 (96.2)	177 (96.2)	
Black	4 (0.9)	0 (0)	
Asian	9 (1.9)	3 (1.6)	
Other	5 (1.1)	4 (2.2)	
Some college or more education, N (%)^b^	375 (85.6)	142 (81.6)	0.140
Married or domestic partnership, N (%)^c^	436 (93.2)	171 (92.9)	0.815
Living situation, N (%)			
Home	431 (91.5)	171 (92.4)	0.437
Skilled care	10 (2.1)	6 (3.2)	
Other	30 (6.4)	8 (4.3)	
Employment status, N (%)^a^			0.339
Full time	39 (8.7)	10 (5.5)	
Part time	27 (6.0)	9 (5.0)	
Not employed outside of home	383 (85.3)	162 (89.5)	
H + Y stage, N (%)^a^			0.002
1 or 2	264 (57.8)	96 (53.0)	
3	140 (30.6)	54 (29.8)	
4 or 5	53 (11.6)	31 (17.1)	
Years since diagnosis, mean (SD)^d^	6.6 (5.1)	7.0 (5.7)	0.760
Number of comorbidities, mean (SD)	3.2 (1.9)	3.3 (1.8)	0.738
Verbal fluency, mean animals/min (SD)^e^	17.0 (6.6)	18.2 (7.1)	0.031
Delayed recall, mean words (SD)^f^	3.4 (1.4)	4.0 (1.3)	0.000
TUG, mean seconds (SD)^g^	14.5 (8.1)	15.3 (8.9)	0.648
Fall frequency, N (%)^a^			0.397
None or rare	342 (73.1)	147 (79.5)	
Weekly or monthly	107 (22.9)	30 (16.2)	
Daily	19 (4.1)	8 (4.3)	
Mean # hospitalizations in past year (SD)	0.8 (1.8)	0.7 (1.1)	0.486
Had ER visit in past year, N (%)^a^	132 (29.5)	50 (27.3)	0.579
Medications^h^			
Levodopa	469 (99.6)	183 (98.9)	0.331
Antidepressant	182 (38.6)	82 (44.3)	0.182
Anticholinergic	17 (3.6)	3 (1.6)	0.183
Cognitive enhancer	89 (18.9)	17 (9.2)	0.002
PDQ-39 summary index, mean (SD)^i^	26.8 (16.0)	28.1 (17.9)	0.592
MDS-UPDRS part II, mean (SD)^j^	28.0 (13.7)	23.9 (14.1)	<0.001

SD: standard deviation; N: number; H + Y: Hoehn and Yahr; TUG: Timed Up & Go test; ER: emergency room; PDQ: Parkinson’s Disease Questionnaire; MDS-UPDRS: Movement Disorder Society-sponsored revision of the Unified Parkinson’s Disease Rating Scale.

*P*-value threshold was kept at 0.05 as an exploratory analysis.

^a^Among patients with data available.

^b^Among patients with data available (174 with male caregivers and 438 with female caregivers).

^c^Among 184 patients with male caregivers and 468 with female caregivers.

^d^Among 185 patients with male caregivers and 468 with female caregivers.

^e^Among 177 patients with male caregivers and 460 with female caregivers.

^f^Delayed recall = number of words recalled from a list of 5.

^g^Among 157 patients with male caregivers and 416 with female caregivers.

^h^There were also no differences in patients with male vs female caregivers taking a monoamine oxidase B inhibitor (*P* = 0.343), a COMT (catechol-O-methyltransferase) inhibitor (*P* = 0.343), a dopamine agonist (*P* = 0.127), or amantadine (*P* = 0.183).

^i^Among 181 patients with male caregivers and 459 with female caregivers.

^j^Among 184 patients with male caregivers and 467 with female caregivers.

### Caregiver Health, Strain, Preparedness, Support, and Satisfaction

Self-reported health is similar between male and female caregivers (*P* = 0.604; *P*-value threshold for significance is 0.003). Female caregivers have more strain (MCSI 19.7 vs 14.8, *P* < 0.001) than men. Caregiver symptoms of depression (CES-D 10.6 vs 8.6, *P* = 0.019) and preparedness (caregiver preparedness score 20.1 vs 21.1, *P* = 0.206) are similar between women and men, respectively. Male and female caregivers have similar access to help from others (*P* = 0.012) and similar feelings of satisfaction from caregiving (*P* = 0.152). However, more female caregivers report having less time for other family members due to caregiving responsibilities (42.7% vs 25.8%, *P* < 0.001) compared to male caregivers (Table 6).

**Table 6.** Caregiver Survey Results Regarding Caregiver Clinical Data and the Caregiving Experience.

Caregiver Data	Female Caregivers (N = 471)	Male Caregivers (N = 185)	*P*-Value
Self-reported health, N (%)^a^			0.604
Good, very good, or excellent	433 (92.1)	171 (92.4)	
Fair or poor	37 (7.9)	14 (7.6)	
CES-D depression score, mean (SD)^b^	10.6 (8.3)	8.6 (6.6)	0.019
Caregiver preparedness score, mean (SD)^c^	20.1 (6.8)	21.1 (6.4)	0.206
MCSI, mean (SD)^d^	19.7 (15.7)	14.8 (13.2)	<0.001
Access to caregiver assistance, N (%)^a^			
Backup caregiver available	219 (48.1)	107 (59.1)	0.012
Has used temporary care, respite or caregiver support services	96 (20.6)	42 (23.0)	0.510
Has participated in support groups	120 (26.1)	47 (26.1)	0.995
Requested financial help information	33 (7.1)	11 (6.0)	0.619
Caregiving experience, N (%)^a^			
Caregiver satisfaction^e^	404 (90.4)	170 (93.9)	0.152
Have less time for other family	195 (42.7)	47 (25.8)	<0.001
Family conflict over caregiving present	161 (35.8)	53 (30.1)	0.383
Costs more than caregiver can afford	55 (12.3)	20 (11.1)	0.670
Changed residence due to patient’s disability	96 (21.1)	27 (14.8)	0.066
Working less because of caregiving	115 (25.1)	38 (20.9)	0.258
Quit job due to caregiving	48 (11.9)	8 (4.8)	0.011

SD: standard deviation; N: number; CES-D: Center for Epidemiologic Studies Depression Scale; MCSI: Multidimensional Caregiver Strain Index.

Corrected *P*-value for statistical significance = 0.003 (using Bonferroni correction accounting for 15 comparisons).

^a^Among caregivers with data available.

^b^Among caregivers with data available (464 women and 185 men).

^c^Among 468 women and 185 men.

^d^Among 395 women and 158 men.

^e^Caregiver satisfaction = answered “agree” or “strongly agree” to “Providing help has made me feel good about myself” or “Providing help has enabled me to appreciate life more”.

## Discussion

This national study examines potential explanations for observed gender differences in caregiving among PwP and shows that the relative lack of informal caregiver support for women with PD reported previously^14^ is largely due to differences in demographics. Women with PD who don’t have caregivers are older and less likely to be married than men without caregivers. Stepwise regressions show that marital status is a strong driver of the gender disparity in caregiver presence.

As opposed to prior research of elderly disabled patients in general that shows wives play a dominant role in the care of disabled men and children (especially daughters, daughters-in-law, or granddaughters) play a dominant role in the care of disabled women,^11^ the vast majority of caregivers in this study for both men and women with PD are spouses of the opposite gender. The high rate of male spouses as the caregiver for the married women with PD in this study may reflect changing demographics and evolving social structures and norms wherein men are increasingly assuming the caregiving role.^26^ This finding may also further magnify the gender disparity in caregiver access in the PD population given that women with PD are less likely to be married and more likely to be widowed, and nearly half of those widowed have no caregiver. Widowhood disproportionately affects women for a variety of reasons; women are more likely to survive the death of their spouse and are less likely to remarry after their spouse dies.^27^

Marriage and widowhood have been shown to affect numerous areas of health. Marriage has a positive impact on the health of older adults in general and widowhood is associated with cognitive decline.^28,29^ In cancer patients, marriage is associated with improved survival and widowhood is associated with the shortest survival rate.^30^ Marriage may affect the health outcomes of men and women with PD differently, as has been shown in cancer patients wherein divorced/separated men have a worse survival rate than women.^30^ This study shows that marriage strongly affects informal caregiver presence in PD, and prior studies indicate that the presence of a caregiver is associated with health outcomes in PD.^9^ It is unknown to what degree marriage and widowhood may independently affect health outcomes in PD and whether the effects of marriage on health outcomes in PD may differ between genders and cultures. This area of research requires further investigation.

Because the PwP in this study are younger than the elderly disabled patients previously studied,^11^ the lower rate of children acting as caregivers for people with PD could reflect the younger age of adult children in this population, who may be busy rearing children and establishing careers, with less time to devote to parental caregiving. Interventions that increase the participation and support of others, including men and women in their support system as well as paid caregivers, in the caregiving for women with PD may help address the relative lack of access to caregivers among women with PD.

The effect of gender differences in caregiving receipt on health outcomes for women with PD requires further investigation. A study of Medicare beneficiaries revealed that women with PD use more advanced nursing care resources (including nursing home placement, home health care, and hospice), have less direct physician contact, and have a higher incidence of hip fracture and depression than men.^31^ Further study is needed to evaluate whether, and to what degree, gender differences in caregiving contribute to these or other adverse healthcare outcomes among women with PD.

Prior studies have also suggested that caregiving has adverse effects on the physical and psychological health of caregivers, particularly among caregivers for dementia patients, including higher rates of depression, stress, lower subjective well-being, and poorer health.^32^ This study shows that female caregivers of PwP report higher rates of strain and have less time to spend with other family members than male caregivers. This may be due to greater cognitive decline among PwP with female caregivers, which has been shown to be more burdensome on caregivers than the motor symptoms of Parkinson’s disease.^33^ Female caregivers also care for PwP with greater disability as evidenced by higher MDS-UPDRS Part II scores, which may contribute to higher caregiver strain. However, this scale could be affected by traditional gender roles; women with PD may score better on the MDS-UPDRS Part II in part because they might be more likely to continue household chores despite their impairment, due to social norms and expectations.^34^ Female caregivers are also more likely to provide care for multiple people, which may reflect traditional gender roles and cultural expectations that shape caregiving responsibilities for women.^15^ Despite this, self-reported health of women caregivers is not poorer than that of men. Addressing gender-specific needs of caregivers will be important for reducing burnout and increasing access to informal caregivers.

There were several limitations to our study. This study is cross-sectional in design, limiting the ability to establish causation. Data gathered for the study relied on self-report, which can lead to bias and missing data. The survey did not capture differences in how men and women caregivers define caregiving, which may be shaped by cultural gender norms. The survey did not account for patients and caregivers with non-binary genders. Participants of the PF-POP may not be representative of the general population, which may affect generalizability of the results. Although the baseline cohort is large, they still represent individuals who are seeking care at Parkinson’s Foundation Centers of Excellence. This cohort is mostly white, with mild disease (H + Y 1 or 2), more educated on average, and may have more resources than the general PD population. Additionally, we relied on a convenience sample of caregivers who chose to respond to the survey which may select for those more interested in or affected by caregiving, thereby creating participation bias. Respondents were mostly spouses of PwP living at home with mild disease, who accompanied the patient to their appointment. These cohort characteristics differ from PwP enrolled in the broader PF-POP who chose not to participate in this sub study (as shown in Table 1) and may differ from the general PD population. These cohort factors limit our ability to understand the caregiving experience of non-spouses and for PwP not living at home. Additionally, the sub-study only captures the experience of the primary caregiver for a patient and does not capture the caregiving experience of any potential secondary caregivers for the same patient. However, the large number of respondents and the detailed nature of the survey allows for the ascertainment of interesting and significant caregiving data that can inform Parkinson’s disease caregiving education and future interventions.

This study highlights the impact of demographic differences between men and women on access to informal caregiving in PD. Identification of factors driving gender disparities in caregiving is important for shaping health policy and implementing interventions to address the unmet needs of both caregivers and PwP without access to informal caregivers. Strategies to detect and address caregiver needs such as systematically screening for caregiver strain and increasing access to social workers are a first step to support existing caregivers. In addition, for all PwP, earlier assessment of needs could help trigger earlier involvement of community and family support networks to identify potential informal caregivers.

## Supplemental Material

Supplemental Material **-** Contributors to Gender Disparities in Parkinson’s Disease Caregiving Supplemental Material for Contributors to Gender Disparities in Parkinson’s Disease Caregiving by Sarah Horn, Yunfeng Dai, Samuel S. Wu, and Nabila Dahodwala in Journal of Geriatric Psychiatry and Neurology.

## Data Availability

The data supporting the findings of this study are available on request from the corresponding author. The data are not publicly available due to privacy or ethical restrictions.*

## References

[1] Hassan A Wu SS Schmidt P , et al. What are the issues facing Parkinson's disease patients at ten years of disease and beyond? data from the NPF-QII study. Parkinsonism Relat Disord. 2012;18(Suppl 3):S10-S14. doi:10.1016/j.parkreldis.2012.06.014 22776044

[2] Antonini A Stoessl AJ Kleinman LS , et al. Developing consensus among movement disorder specialists on clinical indicators for identification and management of advanced Parkinson’s disease: a multi-country delphi-panel approach. Curr Med Res Opin. 2018;34(12):2063-2073. doi:10.1080/03007995.2018.1502165 30016901

[3] Aarsland D Larsen JP Tandberg E Laake K . Predictors of nursing home placement in Parkinson's disease: a population-based, prospective study. J Am Geriatr Soc. 2000;48(8):938-942. doi:10.1111/j.1532-5415.2000.tb06891.x 10968298

[4] Carter JH Stewart BJ Archbold PG , et al. Living with a person who has Parkinson's disease: the spouse's perspective by stage of disease. Parkinson's study group. Mov Disord. 1998;13(1):20-28. doi:10.1002/mds.870130108 9452321

[5] Coelho M Ferreira JJ . Late-stage Parkinson disease. Nat Rev Neurol. 2012;8(8):435-442. doi:10.1038/nrneurol.2012.126 22777251

[6] National Alliance for Caregiving (NAC) and AARP . Caregiving in the U.S. 2020 Report: a focused look at family caregivers of adults age 50+. 2020.

[7] Luppa M Luck T Weyerer S König HH Brähler E Riedel-Heller SG . Prediction of institutionalization in the elderly. A systematic review. Age Ageing. 2010;39(1):31-38. doi:10.1093/ageing/afp202 19934075

[8] Bell JF Whitney RL Young HM . Family caregiving in serious illness in the United States: recommendations to support an invisible workforce. J Am Geriatr Soc. 2019;67(S2):S451-S456. doi:10.1111/jgs.15820 31074854

[9] Prizer LP Kluger BM Sillau S Katz M Galifianakis NB Miyasaki JM . The presence of a caregiver is associated with patient outcomes in patients with Parkinson's disease and atypical parkinsonisms. Parkinsonism Relat Disord. 2020;78:61-65. doi:10.1016/j.parkreldis.2020.07.003 32736164

[10] Navaie-Waliser M Spriggs A Feldman PH . Informal caregiving: differential experiences by gender. Med Care. 2002;40(12):1249-1259. doi:10.1097/01.MLR.0000036408.76220.1F 12458306

[11] Katz SJ Kabeto M Langa KM . Gender disparities in the receipt of home care for elderly people with disability in the United States. JAMA. 2000;284(23):3022-3027. doi:10.1001/jama.284.23.3022 11122589

[12] Luppa M Gentzsch K Angermeyer MC Weyerer S König HH Riedel-Heller SG . Gender-specific predictors of institutionalisation in the elderly--results of the leipzig longitudinal study of the aged (LEILA 75+). Psychiatr Prax. 2011;38(4):185-189. doi:10.1055/s-0030-1248496 20687014

[13] Wong AD Phillips SP . Gender disparities in end of life care: a scoping review. J Palliat Care. 2022;38(1):78-96. doi:10.1177/08258597221120707 35996340 PMC9667103

[14] Dahodwala N Shah K He Y , et al. Sex disparities in access to caregiving in Parkinson disease. Neurology. 2017;90(1):e48-e54. doi:10.1212/WNL.0000000000004764 29196580 PMC10681055

[15] Sharma N Chakrabarti S Grover S . Gender differences in caregiving among family - caregivers of people with mental illnesses. WJP. 2016;6(1):7-17. doi:10.5498/wjp.v6.i1.7 27014594 PMC4804270

[16] Subramanian I Mathur S Oosterbaan A Flanagan R Keener AM Moro E . Unmet needs of women living with Parkinson's disease: gaps and controversies. Mov Disord. 2022;37(3):444-455. doi:10.1002/mds.28921 35060180

[17] Wolff JL Roter DL . Family presence in routine medical visits: a meta-analytical review. Soc Sci Med. 2011;72(6):823-831. doi:10.1016/j.socscimed.2011.01.015 21353358 PMC3070824

[18] Okun MS Siderowf A Nutt JG , et al. Piloting the NPF data-driven quality improvement initiative. Parkinsonism Relat Disord. 2010;16(8):517-521. doi:10.1016/j.parkreldis.2010.06.005 20609611

[19] Radloff LS . The CES-D scale. Appl Psychol Meas. 1977;1(3):385-401. doi:10.1177/014662167700100306

[20] Schrag A Barone P Brown RG , et al. Depression rating scales in Parkinson's disease: critique and recommendations. Mov Disord. 2007;22(8):1077-1092. doi:10.1002/mds.21333 17394234 PMC2040268

[21] Archbold PG Stewart BJ Greenlick MR Harvath T . Mutuality and preparedness as predictors of caregiver role strain. Res Nurs Health. 1990;13(6):375-384. doi:10.1002/nur.4770130605 2270302

[22] Carter JH Lyons KS Stewart BJ Archbold PG Scobee R . Does age make a difference in caregiver strain? Comparison of young versus older caregivers in early-stage Parkinson's disease. Mov Disord. 2010;25(6):724-730. doi:10.1002/mds.22888 20201024

[23] Stull DE . The multidimensional caregiver strain index (MCSI): its measurement and structure. J Clin Geropsychol. 1996;2(3):175-196. Accessed 28 Jan 2020.

[24] Goetz CG Tilley BC Shaftman SR , et al. Movement disorder society-sponsored revision of the unified Parkinson's disease rating scale (MDS-UPDRS): scale presentation and clinimetric testing results. Mov Disord. 2008;23(15):2129-2170. doi:10.1002/mds.22340 19025984

[25] von Elm E Altman DG Egger M , et al. The strengthening the reporting of observational studies in epidemiology (STROBE) statement: guidelines for reporting observational studies. Int J Surg. 2014;12(12):1495-1499. doi:10.1016/j.ijsu.2014.07.013 25046131

[26] Baker KL Robertson N . Coping with caring for someone with dementia: reviewing the literature about men. Aging Ment Health. 2008;12(4):413-422. doi:10.1080/13607860802224250 18791888

[27] Carr D Bodnar-Deren S . Gender, aging and widowhood. In: Uhlenberg P , ed. International Handbook of Population Aging. Netherlands: Springer; 2009:705. doi:10.1007/978-1-4020-8356-3_32

[28] Waldron I Hughes ME Brooks TL . Marriage protection and marriage selection--prospective evidence for reciprocal effects of marital status and health. Soc Sci Med. 1996;43(1):113-123. doi:10.1016/0277-9536(95)00347-9 8816016

[29] Barragán-García M Ramírez-Aldana R López-Ortega M Sánchez-García S García-Peña C . Widowhood status and cognitive function in community-dwelling older adults from the Mexican health and aging study (MHAS). Population Ageing. 2021;15(3):605. doi:10.1007/s12062-020-09322-2 PMC10554834 37800095

[30] Krajc K Miroševič Š Sajovic J , et al. Marital status and survival in cancer patients: a systematic review and meta‐analysis. Cancer Med. 2022;12(2):1685-1708. doi:10.1002/cam4.5003 35789072 PMC9883406

[31] Fullard ME Thibault DP Todaro V , et al. Sex disparities in health and health care utilization after Parkinson diagnosis: rethinking PD associated disability. Parkinsonism Relat Disord. 2018;48:45-50. doi:10.1016/j.parkreldis.2017.12.012. Accessed 2 January 2018. 29273434 PMC6559800

[32] Pinquart M Sörensen S . Differences between caregivers and noncaregivers in psychological health and physical health: a meta-analysis. Psychol Aging. 2003;18(2):250-267. doi:10.1037/0882-7974.18.2.250 12825775

[33] Vatter S Mcdonald KR Stanmore E Clare L Mccormick SA Leroi I . A qualitative study of female caregiving spouses’ experiences of intimate relationships as cognition declines in Parkinson’s disease. Age Ageing. 2018;47(4):604-610. doi:10.1093/ageing/afy049 29617933 PMC6014155

[34] De La Rosa T Amado Scerni D Scorza FA . Sex differences in Parkinson's disease phenotype and caregiving disparities. Mov Disord. 2021;36(2):526. doi:10.1002/mds.28459 33599002

