# Supplementary material for: Contributors to Gender Disparities in Parkinson’s Disease Caregiving
Source: J Geriatr Psychiatry Neurol. 2025 Mar 21;38(5):394–404. doi: 10.1177/08919887251329957 (PMC12202834; doi:10.1177/08919887251329957)
Supplement: Supplemental Material - Contributors to Gender Disparities in Parkinson’s Disease Caregiving [file sj-pdf-1-jgp-10.1177_08919887251329957.pdf]

Parkinson's Outcomes Project
CAREGIVER SUBSTUDY – DATA COLLECTION FORM

NATIONAL PARKINSON FOUNDATION®

**Record identifier**	**Date of visit** MM/DD/YYYY

We thank you for your participation in this survey project. We will ask you information about yourself and your experience providing care for a person with Parkinson's disease (PWP). Please answer each question with the best possible choice listed. Remember that there are no correct answers. The survey takes about 20 minutes to complete.

Basic Information

1	What is your age?	____________ (write in #)	
2	What is your gender?	☐ Male	☐ Female
3	What is your ethnicity?	☐ Hispanic	☐ Non-Hispanic
4	What is your race?	☐ American Indian<br>☐ Asian<br>☐ Pacific Islander<br>☐ Black or African-American<br>☐ White<br>☐ Multiple<br>Write in : ___________________________	
5	Select the highest level of school that you completed:	☐ Less than high school<br>☐ High school graduate<br>☐ Some post-high school education or Associate degree<br>☐ Bachelor's degree<br>☐ Graduate degree (Master's/Professional/Doctoral)	
6	In general, how do your finances usually work out at the end of the month?	☐ Some money left over<br>☐ Just enough to make ends meet<br>☐ Not enough to make ends meet	
7	What is your current marital status?	☐ Single or never married<br>☐ Married or domestic partnership<br>☐ Widowed<br>☐ Divorced or Separated	
8	In general, would you say your health is:	☐ Excellent<br>☐ Very Good<br>☐ Good<br>☐ Fair<br>☐ Poor	

Please answer the following questions about **you and the person with Parkinson's (PWP).**

1	What is **your relationship** to the PWP?	☐ Spouse/ Partner<br>☐ Parent<br>☐ Child<br>☐ Child in-law<br>☐ Other relative<br>☐ Friend<br>☐ Neighbor<br>☐ Other (not relative)
2	Do you **live with the PWP**?<br><br>If **NO**: where does the PWP live?	☐ No<br>☐ Yes<br><br>☐ Lives with spouse/ partner<br>☐ With other family<br>☐ Lives alone<br>☐ Assisted living<br>☐ Independent living<br>☐ Nursing home<br>☐ Other
3	How many children under the age of 18 **live in your household**?	________ (write in #)
4	Do you **provide care for anyone else** other than the PWP?	☐ No<br>☐ Yes
5	How **long ago did you start providing care** because of the PWP's disability?	☐ < 1 year<br>☐ 1 year to < 3 years<br>☐ 3 years to < 6 years<br>☐ > 6 years
6	Do you provide **more care, less care or the same amount of care** now as when you first started providing care?	☐ More<br>☐ Same<br>☐ Less
7	How **long ago did you start providing care as much as you do now**?	☐ < 1 year<br>☐ 1 year to < 3 years<br>☐ 3 years to < 6 years<br>☐ > 6 years
8	On average, **how many days per week** do you provide care?	___ (0-7 days)
9	On the days that you provide care, **how many hours per day** do you provide care?	___ (0-24 hours)

Below is a list of the ways you might have **felt** or **behaved**. Please tell us **how often** you have felt this way during the **past week**.

	**During the past week…**	**Rarely or none of the time** (less than 1 day)	**Some or a little of the time** (1-2 days)	**Occasionally or moderate amount of time** (3-4 days)	**Most or all of the time** (5-7 days)
1	I was bothered by things that usually don't bother me	☐	☐	☐	☐
2	I did not feel like eating; my appetite was poor	☐	☐	☐	☐
3	I felt that I could not shake off the blues even with help from my family or friends	☐	☐	☐	☐
4	I felt I was just as good as other people	☐	☐	☐	☐
5	I had trouble keeping my mind on what I was doing	☐	☐	☐	☐
6	I felt depressed	☐	☐	☐	☐
7	I felt that everything I did was an effort	☐	☐	☐	☐
8	I felt hopeful about the future	☐	☐	☐	☐
9	I thought my life had been a failure	☐	☐	☐	☐
10	I felt fearful	☐	☐	☐	☐
11	My sleep was restless	☐	☐	☐	☐
12	I was happy	☐	☐	☐	☐
13	I talked less than usual	☐	☐	☐	☐
14	I felt lonely	☐	☐	☐	☐
15	People were unfriendly	☐	☐	☐	☐
16	I enjoyed life	☐	☐	☐	☐
17	I had crying spells	☐	☐	☐	☐
18	I felt sad	☐	☐	☐	☐
19	I felt that people dislike me	☐	☐	☐	☐
20	I could not get "going"	☐	☐	☐	☐

Below we list activities for which a person with Parkinson's disease (PWP) might need help, **please check**, **how many times per day** on average do you…

		Never (0)	Seldom (1-2)	Sometimes (3-4)	Often (5-7)
1	Help him/her walk around inside or get around inside with a wheelchair or similar device?	☐	☐	☐	☐
2	Help eat?	☐	☐	☐	☐
3	Help get in and out of bed?	☐	☐	☐	☐
4	Help get dressed by getting and putting on clothes they wear during the day?	☐	☐	☐	☐
5	Give shots or injections?	☐	☐	☐	☐
6	Give medicine, pills or change bandages?	☐	☐	☐	☐
7	Help him/her bathe?	☐	☐	☐	☐
8	Use the toilet by helping him/her get on or off the toilet, arranging clothes or by cleaning him/her?	☐	☐	☐	☐
9	Preparing special foods or fixing extra meals?	☐	☐	☐	☐
10	Managing money, like keeping track of bills or handling cash?	☐	☐	☐	☐
11	Doing things around the house, such as straightening up, putting things away or doing dishes?	☐	☐	☐	☐
12	Doing laundry?	☐	☐	☐	☐
13	Shopping for groceries?	☐	☐	☐	☐
14	Doing other small errands outside of the house?	☐	☐	☐	☐
15	Helping get around outside, including helping walk or use wheelchair or walker?	☐	☐	☐	☐
16	Helping get around the neighborhood or the city by driving or helping use public transportation?	☐	☐	☐	☐

We know that some people may feel well prepared for some aspects of giving care to a person with Parkinson's (PWP), and not as well prepared for other aspects. Please tell us **how well prepared** you think you are to do each of the following even if you are not doing that type of care now.

	How well prepared do you think you are …	Not at all prepared	Not too well prepared	Somewhat prepared	Pretty well prepared	Very well prepared
1	To take care of the PWP's physical needs?	☐	☐	☐	☐	☐
2	To take care of his or her emotional needs?	☐	☐	☐	☐	☐
3	To find out about and set up services for him or her?	☐	☐	☐	☐	☐
4	For the stress of caregiving?	☐	☐	☐	☐	☐
5	To make caregiving activities pleasant for both you and your family members?	☐	☐	☐	☐	☐
6	To respond to and handle emergencies that involve him or her?	☐	☐	☐	☐	☐
7	To get help and information you need from the health care system?	☐	☐	☐	☐	☐
8	Overall, how prepared do you think you are to care for the PWP?	☐	☐	☐	☐	☐
9	Is there anything specific you would like to be better prepared for?<br><br>(Please write in your response)	☐ No	☐ Yes			

____

____

____

____

____

____

____

Please answer the following questions about **receiving help from others**. Pease check the best response.

1	If you were unable to help the PWP, is there someone else who would do the things you do?	☐ No	☐ Yes
2	Have you ever received any respite or caregiver support services from a **government** source to assist you in providing care?	☐ No	☐ Yes
3	Have you ever received any respite or caregiver support services from **another source** to assist you in providing care?	☐ No	☐ Yes
4	Who provided you with this service? (please check all that apply)	☐ Church or synagogue<br>☐ Community or gov't agency<br>☐ Caregiver's employer<br>☐ Individual or private agency<br>☐ Healthcare provider<br>☐ Other<br>☐ N/A	
5	Have you ever taken part in support groups for caregivers?	☐ No	☐ Yes
6	Who provided you with this service?	☐ Church or synagogue<br>☐ Community or gov't agency<br>☐ Caregiver's employer<br>☐ Individual or private agency<br>☐ Healthcare provider<br>☐ Other<br>☐ N/A	
7	Have you ever used a service to temporarily provide care so that you get some time away?	☐ No	☐ Yes
8	Who provided you with this service?	☐ Church or synagogue<br>☐ Community or gov't agency<br>☐ Caregiver's employer<br>☐ Individual or private agency<br>☐ Healthcare provider<br>☐ Other<br>☐ N/A	
9	Have you ever requested information about how to get financial help?	☐ No	☐ Yes

Please answer the following questions about **your caregiving experience**. Please check the best response.

1	Providing help has made me feel good about myself.	☐ Strongly Agree<br>☐ Agree<br>☐ Disagree<br>☐ Strongly Disagree
2	Providing help has enabled me to appreciate life more.	☐ Strongly Agree<br>☐ Agree<br>☐ Disagree<br>☐ Strongly Disagree
3	To what extent has there been any family conflict over caregiving?	☐ A lot of conflict<br>☐ Some conflict<br>☐ None at all
4	Does providing care cost more than you can really afford?	☐ No ☐ Yes
5	As a caregiver, have you had less time for other family members than before?	☐ No ☐ Yes
6	Have you ever changed your place of residence because of the PWP's disability?	☐ No ☐ Yes
7	Are you currently working for pay at a job or business?	☐ No ☐ Yes
8	How many hours per week do you usually work?	___________(fill in # of hours)
9	Are you working fewer hours than you would like to because you help provide care	☐ No ☐ Yes ☐ N/A
10	If you stopped working, what was the main reason?	☐ Retired<br>☐ Sick/disabled<br>☐ Had to provide care<br>☐ Fired/Laid off<br>☐ Other<br>☐ Not applicable- I am still working
11	If you stopped working, would you have continued working longer if you were not providing care?	☐ No ☐ Yes ☐ N/A

We are interested in the **average or usual function** of the person with Parkinson's disease (PWP) **over the past week including today**. Some PWP can do things better at one time of the day than at others. However, only one answer is allowed for each question, so please mark the answer that best describes what he or she can do most of the time. Do not worry about separating Parkinson's disease from other conditions. **Please choose the best response.**

Over the past week has the person with Parkinson's disease (PWP) had trouble…

1 **Going to sleep at night or staying asleep through the night?** Consider how rested he/she felt after waking up in the morning. [sleep problems]

- ☐ **Normal**: No problems.
- ☐ **Slight**: Sleep problems are present but usually do not cause trouble getting a full night of sleep.
- ☐ **Mild**: Sleep problems usually cause some difficulties getting a full night of sleep.
- ☐ **Moderate**: Sleep problems cause a lot of difficulties getting a full night of sleep, but PWP still usually sleeps for more than half the night.
- ☐ **Severe**: PWP usually does not sleep for most of the night.

2 **Staying awake during the daytime?** [daytime sleepiness]

- ☐ **Normal**: No daytime sleepiness.
- ☐ **Slight**: Daytime sleepiness occurs but PWP can resist and stay awake.
- ☐ **Mild**: Sometimes PWP falls asleep when alone and relaxing. For example, while reading or watching TV.
- ☐ **Moderate**: PWP sometimes falls asleep when he/she should not. For example, while eating or talking with other people.
- ☐ **Severe**: PWP often falls asleep when he/she should not. For example, while eating or talking with other people.

3 **Uncomfortable feelings in PWP's body like pain, aches tingling or cramps?** [pain]

- ☐ **Normal**: No uncomfortable feelings.
- ☐ **Slight**: PWP has these feelings. However, he/she can do things and be with other people without difficulty.
- ☐ **Mild**: These feelings cause some problems when he/she does things or is with other people.
- ☐ **Moderate**: These feelings cause a lot of problems, but does not stop him/her from doing things or being with other people.
- ☐ **Severe**: These feelings stop him/ from doing things or being with other people.

Over the past week has the person with Parkinson's disease (PWP) had trouble…

4 **With urine control**? For example, an urgent need to urinate, a need to urinate too often, or urine accidents? [urinary problems]

- ☐ **Normal**: No urine control problems.
- ☐ **Slight**: PWP needs to urinate often or urgently. However, these problems do not cause difficulties with daily activities.
- ☐ **Mild**: Urine problems cause some difficulties with his/her daily activities. However, PWP does not have urine accidents.
- ☐ **Moderate**: Urine problems cause a lot of difficulties with daily activities, including urine accidents.
- ☐ **Severe**: PWP cannot control urine and uses a protective garment or has a bladder tube.

5 **Troubles that cause PWP difficulty moving bowels?** [constipation problems]

- ☐ **Normal**: No constipation.
- ☐ **Slight**: PWP has been constipated. He/she uses extra effort to move bowels. However, this problem does not disturb activities or being comfortable.
- ☐ **Mild**: Constipation causes him/her to have some troubles doing things or being comfortable. However, PWP does not have urine accidents.
- ☐ **Moderate**: Constipation causes him/her to have a lot of trouble doing things or being comfortable. However, it does not stop him/her from doing anything.
- ☐ **Severe**: PWP usually needs physical help from someone else to empty bowels.

6 **Felt faint, dizzy or foggy when PWP stands up after sitting or lying down?** [light headed]

- ☐ **Normal**: No dizzy or foggy feelings.
- ☐ **Slight**: Dizzy or foggy feelings occur. However, feelings do not cause any troubles doing things.
- ☐ **Mild**: Dizzy or foggy feelings cause PWP to hold on to something, but he/she does not need to sit or lie back down.
- ☐ **Moderate**: Dizzy or foggy feelings cause him/her to sit or lie down to avoid fainting or falling.
- ☐ **Severe**: Dizzy or foggy feelings cause him/her to fall or faint.

Over the past week has the person with Parkinson's disease (PWP) had trouble…

7 **Felt fatigued?** This feeling is not part of being sleepy or sad. [fatigue]

- ☐ **Normal**: No fatigue.
- ☐ **Slight**: Fatigue occurs. However, it does not cause any troubles doing things or being with people.
- ☐ **Mild**: Fatigue causes him/her some troubles doing things or being with people.
- ☐ **Moderate**: Fatigue causes him/her a lot of troubles doing things or being with people. However, it does not stop him/her from doing anything.
- ☐ **Severe**: Fatigue stops him/her from doing things or being with people.

8 **Problems with speech?** [speech]

- ☐ **Normal**: No problems.
- ☐ **Slight**: PWP's speech is soft, slurred or uneven, but it does not cause others to ask him/her to repeat self.
- ☐ **Mild**: PWP's speech causes people to ask him/her to occasionally repeat self, but not every day.
- ☐ **Moderate**: PWP's speech is unclear enough that others ask him/her to repeat self every day even though most of his/her speech is understood.
- ☐ **Severe**: Most or all of his/her speech cannot be understood.

9 **Too much saliva when PWP is awake or asleep?** [saliva]

- ☐ **Normal**: No problems.
- ☐ **Slight**: PWP has too much saliva, but does not drool.
- ☐ **Mild**: PWP has some drooling during sleep, but none when he/she is awake.
- ☐ **Moderate**: PWP has some drooling when awake, but usually does not need tissues or a handkerchief.
- ☐ **Severe**: PWP has so much drooling that he/she regularly needs to use tissues or a handkerchief to protect clothes.

Over the past week has the person with Parkinson's disease (PWP) had trouble…

10 **Swallowing pills or eating meals?** Does PWP need pills cut or crushed or meals to be made soft, chopped or blended to avoid choking? [chewing and swallowing]

- ☐ **Normal**: No problems.
- ☐ **Slight**: PWP is aware of slowness in chewing or increased effort at swallowing, but he/she does not choke or need to have food specially prepared.
- ☐ **Mild**: PWP needs to have pills cut or food specially prepared because of chewing or swallowing problems, but he/she has not choked over the past week.
- ☐ **Moderate**: PWP choked at least once in the past week.
- ☐ **Severe**: Because of chewing and swallowing problems, PWP needs a feeding tube.

11 **Handling food and using eating utensils?** For example, does PWP have trouble handling finger foods or using forks, knifes, spoons, chopsticks? [eat]

- ☐ **Normal**: No problems.
- ☐ **Slight**: PWP is slow, but does not need any help handling food and has not had food spills while eating.
- ☐ **Mild**: PWP is slow with eating and have occasional food spills. He/she may need help with a few tasks such as cutting meat.
- ☐ **Moderate**: PWP needs help with many eating tasks but can manage some alone.
- ☐ **Severe**: PWP needs help for most or all eating tasks.

12 **Dressing?** For example, is PWP slow or needs help with buttoning, using zippers, putting on or taking off clothes or jewelry? [dressing]

- ☐ **Normal**: No problems.
- ☐ **Slight**: PWP is slow but does not need help.
- ☐ **Mild**: PWP is slow and needs help for a few dressing tasks (buttons,bracelets).
- ☐ **Moderate**: PWP needs help for many dressing tasks.
- ☐ **Severe**: PWP needs help for most or all dressing tasks.

Over the past week has the person with Parkinson's disease (PWP) had trouble…

13 **Being slow or does PWP need help with washing, bathing, shaving, brushing teeth, combing hair or with other personal hygiene?** [hygiene]

- ☐ **Normal**: No problems.
- ☐ **Slight**: PWP is slow but does not need help.
- ☐ **Mild**: PWP needs someone else to help with some hygiene tasks.
- ☐ **Moderate**: PWP needs help for many hygiene tasks.
- ☐ **Severe**: PWP needs help for most or all of their hygiene tasks.

14 **Reading PWP's handwriting?** [handwriting]

- ☐ **Normal**: No problems.
- ☐ **Slight**: PWP's writing is slow, clumsy or uneven, but all words are clear.
- ☐ **Mild**: Some words are unclear and difficult to read.
- ☐ **Moderate**: Many words are unclear and difficult to read.
- ☐ **Severe**: Most or all words cannot be read.

15 **Doing hobbies or other things that PWP likes to do?** [hobbies]

- ☐ **Normal**: No problems.
- ☐ **Slight**: PWP is a bit slow but does these activities easily.
- ☐ **Mild**: PWP has some difficulty doing these activities.
- ☐ **Moderate**: PWP has major problems doing these activities, but still does most.
- ☐ **Severe**: PWP is unable to do most or all of these activities.

16 **Turning over in bed?** [turning on bed]

- ☐ **Normal**: No problems.
- ☐ **Slight**: PWP has a bit of trouble turning, but does not need any help.
- ☐ **Mild**: PWP has a lot of trouble turning and needs occasional help from someone else.
- ☐ **Moderate**: To turn over he/she often needs help from someone else.
- ☐ **Severe**: PWP is unable to turn over without help from someone else.

Over the past week has the person with Parkinson's disease (PWP) had trouble…

17 **Shaking or tremor?** [tremor]

- ☐ **Normal**: Not at all. PWP has no shaking or tremor.
- ☐ **Slight**: Shaking or tremor occurs but does not cause problems with any activities.
- ☐ **Mild**: Shaking or tremor causes problems with only a few activities.
- ☐ **Moderate**: Shaking or tremor causes problems with many of his/her daily activities.
- ☐ **Severe**: Shaking or tremor causes problems with most or all activities.

18 **Getting out of bed, a car seat, or a deep chair?** [getting out of bed]

- ☐ **Normal**: No problems.
- ☐ **Slight**: PWP is slow or awkward, but usually can do it on his/her first try.
- ☐ **Mild**: PWP needs more than one try to get up or needs occasional help.
- ☐ **Moderate**: PWP sometimes needs help to get up, but most times can still do it on his/her own.
- ☐ **Severe**: PWP needs help most or all of the time.

19 **Balance and walking?** [walking and balance]

- ☐ **Normal**: No problems.
- ☐ **Slight**: PWP is slightly slow or may drag a leg. He/she never uses a walking aid.
- ☐ **Mild**: PWP occasionally uses a walking aid, but does not need any help from another person.
- ☐ **Moderate**: PWP usually uses a walking aid (cane, walker) to walk safely without falling. However, he/she does not usually need the support of another person.
- ☐ **Severe**: PWP usually uses the support of another person to walk safely without falling.

Over the past week has the person with Parkinson's disease (PWP) had trouble…

20 On a usual day when walking, does PWP suddenly stop or freeze as if feet are stuck to the floor**?** [freezing]

- ☐ **Normal**: No problems.
- ☐ **Slight**: PWP briefly freezes but can easily start walking again. He/she does not need help from someone else or a walking aid (cane or walker) because of freezing.
- ☐ **Mild**: PWP freezes and has trouble starting to walk again, but does not need someone's help or a walking aid (cane or walker) because of freezing.
- ☐ **Moderate**: When PWP freezes, he/she has a lot of trouble starting to walk again and, because of freezing, sometimes need to use a walking aid or need someone else's help.
- ☐ **Severe**: Because of freezing, most or all of the time, PWP needs to use a walking aid or someone's help.

You are now finished with the caregiving survey project. We thank you for your time and thoughtful consideration of these questions. Your answers will help us develop better programs to support caregiving in Parkinson's disease.
